# Cellular Release of Infectious Hepatitis C Virus Particles via Endosomal Pathways

**DOI:** 10.3390/v15122430

**Published:** 2023-12-14

**Authors:** Lin Deng, Muchamad Ridotu Solichin, Dewa Nyoman Murti Adyaksa, Maria Alethea Septianastiti, Rhamadianti Aulia Fitri, Gede Ngurah Rsi Suwardan, Chieko Matsui, Takayuki Abe, Ikuo Shoji

**Affiliations:** 1 Division of Infectious Disease Control, Center for Infectious Diseases, Kobe University Graduate School of Medicine, Kobe 650-0017, Japan; denglm@med.kobe-u.ac.jp (L.D.); dewa.murti.a@mail.ugm.ac.id (D.N.M.A.); marialethea@gmail.com (M.A.S.); atakayu@med.kobe-u.ac.jp (T.A.); 2 Faculty of Medicine, Public Health, and Nursing, Universitas Gadjah Mada, Yogyakarta 55281, Indonesia; 3 Department of Clinical Microbiology, Faculty of Medicine, Universitas Udayana, Bali 80361, Indonesia

**Keywords:** hepatitis C virus, release, Golgi, recycling endosome, multivesicular bodies, endosomal sorting complex required for transport machinery

## Abstract

Hepatitis C virus (HCV) is a positive-sense, single-stranded RNA virus that causes chronic hepatitis, liver cirrhosis and hepatocellular carcinoma. The release of infectious HCV particles from infected hepatocytes is a crucial step in viral dissemination and disease progression. While the exact mechanisms of HCV particle release remain poorly understood, emerging evidence suggests that HCV utilizes intracellular membrane trafficking and secretory pathways. These pathways include the Golgi secretory pathway and the endosomal trafficking pathways, such as the recycling endosome pathway and the endosomal sorting complex required for transport (ESCRT)-dependent multivesicular bodies (MVBs) pathway. This review provides an overview of recent advances in understanding the release of infectious HCV particles, with a particular focus on the involvement of the host cell factors that participate in HCV particle release. By summarizing the current knowledge in this area, this review aims to contribute to a better understanding of endosomal pathways involved in the extracellular release of HCV particles and the development of novel antiviral strategies.

## 1. Introduction

According to the World Health Organization, an estimated 58 million people worldwide are chronically infected with hepatitis C virus (HCV) and 1.5 million new infections occur each year. HCV remains a significant public health burden [1]. HCV is a leading cause of chronic hepatitis, liver cirrhosis, and hepatocellular carcinoma [2]. Although there have been advancements in direct-acting antiviral (DAA) therapy, which can cure over 95% of patients infected with HCV, the emergence of resistance-associated substitutions (RASs) and limited access to DAAs therapy in developing countries hinder global HCV elimination efforts [3,4,5]. 

HCV is an enveloped, positive-sense single-stranded RNA virus that belongs to the *Hepacivirus* genus of the *Flaviviridae* family. The HCV genome consists of 9.6-kb RNA encoding a single polyprotein of approximately 3010 amino acids, which is processed by viral proteases and cellular signalases to produce three structural proteins (Core, E1, and E2) and seven nonstructural proteins (p7, NS2, NS3, NS4A, NS4B, NS5A, and NS5B) [6,7,8]. The structural proteins are incorporated into virus particles, while the nonstructural (NS) proteins are not incorporated into virus particles. NS proteins are essential for HCV RNA replication and virus assembly [9]. 

HCV particles circulate in the blood and exhibit a strict liver tropism contacting with the basolateral side of hepatocytes. HCV particles attach to the cell surface via glycosaminoglycans, low-density lipoprotein receptor, and scavenger receptor B1. This is followed by binding of E1-E2 dimers to CD81 and Claudin-1, which is in contact with Occludin. HCV particles are then engulfed by clathrin-mediated endocytosis and fused with the endoplasmic membrane under low-pH conditions [10]. The HCV genomic RNA is released into the cytoplasm, where the HCV genomic RNA is directly translated to produce viral proteins and initiate viral replication [9,11]. 

HCV RNA replication occurs within the membranous web on the endoplasmic reticulum (ER) membrane [7,12,13]. During virus particle assembly, viral RNA is shifted away from replication or translation and directed toward the assembly site at the ER membrane, where viral RNA associates with the core protein. NS5A and NS3/4A are involved in transferring RNA from the replication site to the assembly site [14,15,16]. The interaction between NS2 and NS3 is essential for recruiting the viral core protein from lipid droplets to the assembly site, leading to the formation of the nucleocapsid [17]. Nascent virus particles assemble through the recruitment of E1-E2 complexes and bud into the ER lumen. In the ER, the virus particles may fuse with very-low-density lipoproteins, resulting in the formation of mature lipoviroparticles (LVPs) [9,18]. 

The release of HCV particles is the final step of the HCV life cycle. HCV is known to be released via two distinct routes: extracellular release and cell-to-cell transmission [19,20]. In this review, we focus on the pathways involved in extracellular release. HCV has evolved to exploit intracellular membrane trafficking machineries for its release from the cells. While the ER-*trans*-Golgi network (TGN)-recycling endosomal secretion pathway has been considered as the canonical route for HCV particle release [21,22,23], recent research reports have demonstrated the involvement of the endosomal sorting complex required for the transport (ESCRT)-dependent multivesicular bodies (MVBs) secretory pathway in HCV release [24,25,26]. In addition to the primary routes (ER-TGN-recycling endosomal pathway and ER-TGN-MVB pathway), HCV also utilizes unconventional secretion pathways for particle release. These pathways include the autophagy-related release pathway [27,28], and the ER-to-Golgi bypass pathway, in which HCV-induced ER stress triggers HCV release, bypassing the Golgi directly [28,29,30]. In this review, we focus on the host factors involved in HCV particle release via the pathways of ER-TGN-recycling endosome and ER-TGN-MVB. 

## 2. Transport of HCV Particles from the ER to the Golgi Apparatus in Coat Protein Complex II (COPII)-Coated Vesicles

### 2.1. COPII Vesicles and Rab1 GTPases

The biogenesis of most cellular membrane proteins and secreted proteins occurs at the ER. These proteins are transported from the ER to the Golgi apparatus via the COPII vesicle machinery and finally redistributed to their final destinations [31,32]. The COPII coat machinery consists of five cytosolic proteins: Sar1, Sec23, Sec24, Sec13, and Sec31. Sar1 is the first COPII component recruited to the ER membrane. Sec23 and Sec24 form the inner COPII coat, while Sec13 and Sec31 form the outer COPII coat. Sar1, the inner-coat complex, and the outer-coat complex assemble together to form a complete COPII vesicle, which buds from the ER through membrane fission [33,34,35].

In mammalian cells, the directionality of COPII vesicle transport is mediated by Rab1 GTPases, which belong to the Rab GTPase family. Rab GTPase family is a part of the Ras superfamily of small GTPases, consisting of at least 60 members in humans. Rab1 has two isoforms, Rab1a and Rab1b, which localize in the ER-Golgi interface and participate in the COPII-dependent ER-to-Golgi transport [36,37,38,39,40,41].

### 2.2. Transport of HCV Particles via ER-to-Golgi Trafficking 

**Figure 1:**
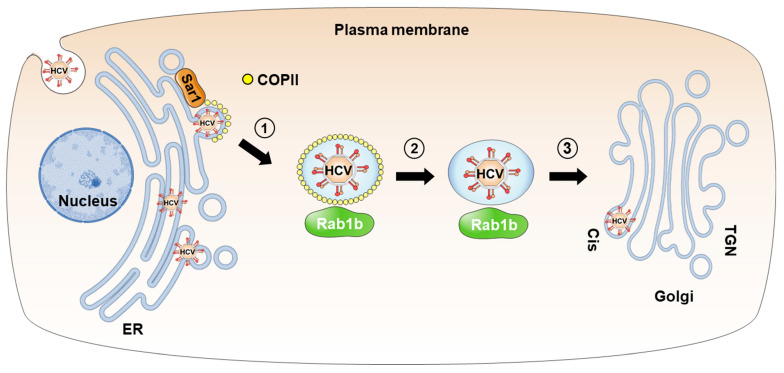
Transport of HCV particles from the ER to the Golgi apparatus in COPII-coated vesicles. HCV particles are assembled in the ER and then bud into COPII-coated vesicles (step 1). Rab1b mediates trafficking of vesicles from ER to Golgi. Following budding, the COPII coat is removed (step 2), and the vesicle is transported to the *cis*-Golgi (step 3), where docking is followed by the delivery of HCV particles. Sar1 is a COPII component.

HCV RNA is enriched in highly buoyant COPII vesicle fractions, cofractionated with apolipoprotein B (ApoB), ApoE, and the HCV core and envelope proteins E1/E2. Moreover, electron microscopy and ultrastructural analysis revealed that HCV envelope and core proteins were colocalized with apolipoproteins and HCV RNA in the Sec31-coated COPII vesicles, as well as in the Golgi stacks [23]. An analysis of the dynamics of HCV core trafficking in Huh-7.5 cells revealed that HCV core proteins were colocalized with Golgi markers [22], supporting the notion that HCV particles are assembled in the ER and transported to the Golgi apparatus in COPII vesicles (Figure 1). The presence of HCV particles in COPII vesicles is further supported by the observation that the reduced expression of Sar1 results in the retention of viral particles in infected cells [22].

Additionally, inhibition of Rab1b reduces the release of HCV particles, suggesting that ER-to-Golgi trafficking participates in the transport of HCV particles [42]. 

## 3. Transport of HCV Particles from the Golgi Apparatus to Recycling Endosomes

### 3.1. Golgi Apparatus and Cellular Proteins Required for Transport from Golgi to Recycling Endosomes

The Golgi apparatus plays a central role in the secretory pathway, serving as a hub for vesicle trafficking. The Golgi stack consists of three compartments: *cis*, *medial*, and *trans* compartments. At the *cis*-face, a collection of vesicular tubular clusters mediates transport between the ER and the Golgi stack. At the *trans*-face, the TGN acts as a major sorting station for the secretory pathway and represents the final exit to the cell surface [43]. The TGN receives proteins that have passed through the Golgi stack and distributes them to various cellular locations, including the plasma membrane, secretory vesicles, and endosomes [44,45,46]. The formation of vesicles from the TGN, directed towards their destinations is facilitated by coat and adaptor proteins, actin, and the microtubule cytoskeleton [47,48]. 

Endosomal trafficking is an essential cell process involved in the transport of proteins and lipids. There are three different types of endosomes: early endosomes, late endosomes, and recycling endosomes. The early endosomes mature into late endosomes, also known as MVBs. The recycling endosomes are concentrated at the microtubule organizing center and consist of a mainly tubular network [49]. Both recycling endosomes and MVBs serve as intermediates during protein transport from the Golgi to the plasma membrane [50,51].

Clathrin, a vesicle coat protein, is involved in the formation of vesicles from the plasma membrane, endosomal membranes, and TGN by inducing membrane curvature [52,53]. The heterotetrameric adaptor protein complexes (APs) act as major cytosolic cargo adaptors, with five APs identified in eukaryotes. Two of these adaptors, AP-1 and AP-4, are involved in protein sorting at the TGN. AP-1 is a clathrin-dependent adaptor, while AP-4 is not associated with clathrin. AP-1 has two isoforms, AP-1A and AP-1B. AP-1A primarily regulates sorting from the TGN to the recycling endosome, and AP-1B regulates sorting from the TGN to the basolateral plasma membrane. AP-4 is involved in sorting and exiting from the TGN to both the endosomal and basolateral pathways [54,55,56,57].

Other clathrin-associated adaptors are Golgi-localized, gamma adaptin ear-containing, and ARF-binding (GGA) proteins: GGA1, GGA2, and GGA3. These proteins mediate vesicular transport between the TGN and endosomes [58].

Rab11A and Rab13, members of the Rab protein family, participate in the membrane traffic pathway from the TGN to recycling endosomes before being delivered to the plasma membrane [39,59,60].

### 3.2. Transport of HCV Particle via TGN-to-Recycling Endosomes Trafficking

**Figure 2:**
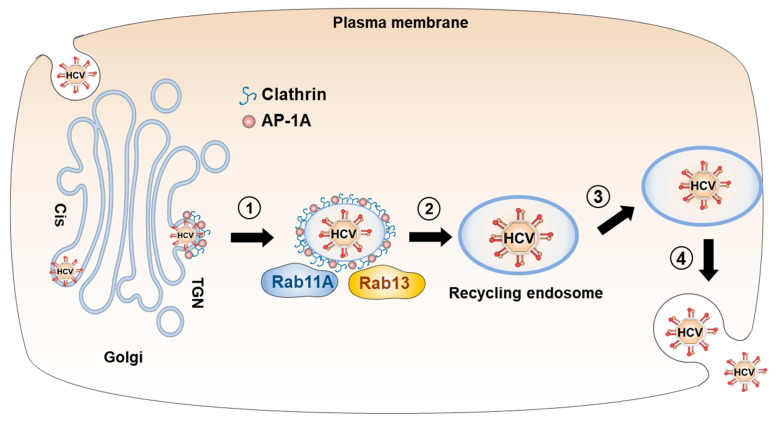
Transport of HCV particles from the Golgi apparatus to recycling endosome. HCV particles egress from the *trans*-Golgi network (TGN) into clathrin/AP-1-coated vesicles (step 1). Rab11A and Rab13 mediate trafficking of clathrin/AP-1-coated vesicles to recycling endosome (step 2). Upon reaching the plasma membrane (step 3), HCV particles are released via exocytosis following fusion with the plasma membrane (step 4).

The release of HCV particles depends on several components of the TGN-to-recycling endosome pathway (Figure 2). Accumulating evidence suggests that HCV exploits AP complexes to facilitate the trafficking of HCV particles during the release. Knockdown of clathrin or the clathrin adaptor AP-1 [61], AP-1 μ1 subunit [21,22] or γ subunit [61] in HCV-infected hepatocytes decrease extracellular infectivity titers without altering intracellular infectivity titers. Additionally, two dileucine-based motifs in the C-terminus of the HCV NS2 protein mediate the binding to AP-1A, AP-1B, and AP-4. AP-1A is involved in HCV particle release, while AP-1B and AP-4 mediate cell-to-cell spread [62], further supporting the requirement of AP-1A for HCV release [22,61,63]. 

Silencing of GGA2 mRNA reduces extracellular infectivity, indicating that GGA2, but not GGA1 or GGA3, is necessary for viral release [21].

Silencing of Rab11A mRNA leads to the accumulation of HCV core protein at the Golgi, indicating that HCV particles are released from the TGN to recycling endosomes and subsequently to the plasma membrane [22]. Additionally, the dominant negative Rab13 protein inhibits the release of infectious HCV [21], further suggesting that infectious HCV particles exploit TGN-to-recycling endosomes trafficking before being released into the extracellular space. 

## 4. Transport of HCV Particles from the Golgi Apparatus to MVBs

### 4.1. MVB Biogenesis and ESCRT Machinery

MVBs are crucial components in endosomal trafficking. MVBs can fuse with lysosomes, resulting in the degradation of their contents. Additionally, MVBs can be directed towards the plasma membrane, where MVBs fuse with the plasma membrane, releasing the contents outside the cell, known as exosomes [51]. MVB formation is regulated by ESCRT machinery, which facilitates membrane abscission processes and intraluminal budding on endosomal membranes. The ESCRT machinery comprises five protein complexes (ESCRT-0, -I, -II, -III, and VPS4) along with associated proteins. These complexes function sequentially to recruit and cluster cargo proteins (ESCRT-0), induce membrane curvature (ESCRT-I and -II), and catalyze vesicle fission (ESCRT-III and VPS4). The AAA ATPase VPS4 (two isoforms, VPS4A and VPS4B) eventually disassembles the ESCRT-III complex from the MVB membrane, thereby driving membrane fission and recycling of ESCRT-III subunits [64,65].

### 4.2. Transport of HCV Particle via TGN-to-MVB Trafficking

**Figure 3:**
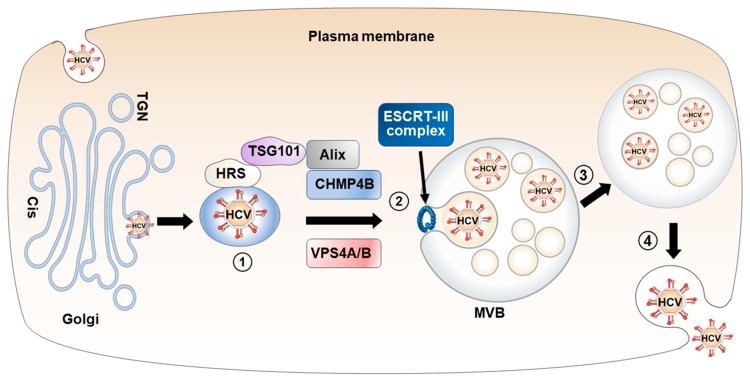
Transport of HCV particles from the Golgi apparatus to MVBs. HCV proteins interact with HRS, an ESCRT-0 component, facilitating the entry of HCV particles into the ESCRT network (step 1). HCV particles are directed into the MVBs through the involvement of key components such as TSG101 (an ESCRT-1 component), CHMP4B (an ESCRT-III component), Alix (an accessory protein that binds to TSG101 and CHMP4B), and VPS4A/B (step 2). MVBs approach the plasma membrane (step 3), HCV particles are released via exocytosis after fusing with the plasma membrane (step 4).

HCV release depends on several components of the ESCRT (Figure 3). Dominant-negative forms of VPS4 or CHMP4B, a component of ESCRT-III, have been found to reduce the release of HCV particles without affecting the intracellular virus titers [26]. Similarly, knockdown of VPS4B, CHMP4B, TSG101 (a component of ESCRT-1), and Alix (an accessory protein that binds to TSG101 and CHMP4B) mRNAs inhibits the release of HCV particles without affecting HCV replication and intracellular infectivity [25,66]. These findings support the requirement of ESCRT for the release of infectious HCV particles, without directly impacting HCV replication or assembly. Electron microscopy analysis also revealed the presence of HCV particles, HCV core protein, and envelope proteins within the intraluminal vesicles of MVBs [67,68,69]. Additionally, the HCV core protein in the supernatants of HCV-infected cells was localized to the exosome-rich fractions [69], indicating the utilization of exosome secretion during HCV release. HCV NS2 and NS5A proteins interact with HRS, an ESCRT-0 component, and utilize HRS as an entry point into the ESCRT network [66].

### 4.3. HCV-Induced ROS/JNK/Itch Signaling Pathway Promotes VSP4A Polyubiquitylation, Leading to Enhancement of VPS4A ATPase Activity, Thereby Upregulating the Release of HCV Particles

**Figure 4:**
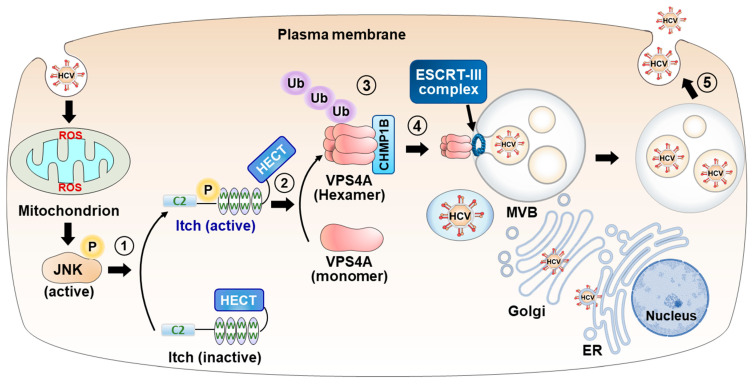
HCV-induced ROS/JNK/Itch signaling pathway promotes VSP4A polyubiquitylation, leading to enhancement of VPS4A ATPase activity, thereby upregulating the release of HCV particles. HCV infection induces mitochondrial ROS production and JNK activation, which phosphorylates Itch, a HECT-type E3 ubiquitin ligase, causing a conformational change and activation of Itch (step 1). Activated Itch promotes the polyubiquitylation of VPS4A (step 2), increasing its interaction with CHMP1B (step 3), which is involved in the promotion of VPS4A ATPase activity and formation of a VPS4A hexamer. Activated VPS4A dissociates ESCRT-III complex from endosomal membranes (step 4), resulting in membrane scission and formation of the MVB. Subsequently, ESCRT-MVB pathway-mediated HCV particle release is enhanced (step 5). P, phosphorylation; Ub, ubiquitylation.

While the studies mentioned above demonstrate the involvement of VPS4 in the release of HCV particles, the precise mechanisms underlying how HCV exploits VPS4 through activating VPS4 ATPase activity were investigated. VPS4 ATP hydrolysis is required for the disassembly of the ESCRT-III complex from the MVB membrane [70]. The interaction between VPS4 and ESCRT-III proteins leads to the induction of VPS4 ATPase activity through the relief of VPS4 autoinhibition [71,72]. Our laboratory recently reported that HCV-induced reactive oxygen species (ROS)/c-Jun N-terminal kinase (JNK)/Itch signaling pathway promotes VPS4A polyubiquitylation, leading to the enhancement of VPS4A ATPase activity, thereby upregulating the release of HCV particles [24] (Figure 4). 

We and other groups previously reported that HCV infection induces mitochondrial ROS production and activates the JNK signaling pathway [73,74,75,76]. Itch is a member of the neural precursor cell-expressed developmentally downregulated protein 4 (NEDD4) family of HECT-type E3 ligases [77,78]. Under physiological conditions, Itch WW domains restrict the interdomain mobility of the HECT domain, locking Itch in a closed inactive conformation [79]. Activated JNK phosphorylates Itch at Ser199, Thr222, and Ser232, leading to a conformational change that weakens the interaction between the WW and HECT domains, thereby enhancing the catalytic activity of Itch [80]. 

In our study, we first demonstrated that HCV infection promotes phosphorylation of Itch at Thr222 via the ROS/JNK signaling pathway. Furthermore, knockdown of Itch does not affect HCV replication but decreases the release of HCV particles, indicating the involvement of Itch in HCV release. Cell-based ubiquitylation assays showed that HCV-induced JNK/Itch signaling pathway specifically promotes polyubiquitylation of VPS4A, but not VPS4B. Additionally, VPS4A, but not VPS4B, is involved in the release of HCV particles. We explored the impact of the VPS4A polyubiquitylation on the activation of VPS4A ATPase activity. Immunoprecipitation analysis revealed that HCV infection specifically enhances the interaction between VPS4A and CHMP1B, a component of ESCRT-III, via VPS4A polyubiquitylation. Moreover, HCV infection significantly enhances ATPase activity of VPS4A, but not VPS4B. Our results clearly demonstrated that the ROS/JNK/Itch signaling pathway enhances the release of HCV particles via the polyubiquitylation of VPS4A [24]. 

Notably, Itch is also exploited by other RNA viruses and DNA viruses for viral release or budding. For example, Itch induces the release of influenza A virus from endosomes through the ubiquitylation of the viral M1 protein [81]. Itch also facilitates Ebola virus budding [82] and nuclear egress of the Epstein–Barr virus [83] through interaction with viral proteins VP40 and BFRF1, respectively. Knockdown of Itch expression reduces the release of human T-cell leukemia virus type 1 [84]. These findings highlight the importance of the E3 ligase Itch in viral release or budding. 

Viruses typically recruit the ESCRT machinery through the late domains, which are conserved motifs found within viral structural proteins. Some characterized late domains include P(T/S)AP, YPXL, and PPXY signals (where X is any amino acid), which bind to TSG101 (ESCRT-1), Alix, or E3 ligase NEDD4 family proteins [85]. Although HCV structural and nonstructural proteins lack defined late domains [25,66], further investigations are needed to clarify whether Itch participates in the ubiquitylation of HCV structural proteins, which could play a role in the process of entry into the ESCRT machinery. 

Additionally, some enveloped RNA viruses, like human immunodeficiency virus type 1, employ the ESCRT machinery to obtain their membrane envelopes, influencing their assembly and release following membrane scission [86]. ESCRT machinery is also exploited by nonenveloped RNA viruses, such as hepatitis A virus [87], bluetongue virus [88], and enveloped DNA viruses, including hepatitis B virus [89], to aid in intracellular budding or release.

## 5. Conclusions

In Table 1, we summarized host factors that participate in HCV particle release, which are discussed in this review. In this review, our emphasis is on the utilization of intracellular membrane trafficking machineries for HCV particle release, spanning from the ER to the Golgi, through the endosomes (recycling endosomes or MVB), and ultimately reaching the plasma membrane. Despite these detailed investigations, the precise mechanisms directing HCV particle sorting into recycling endosomes or MVB remain unclear. Notably, HCV employs alternative release pathways, including cell–cell transmission, the autophagy-related release pathway, and the ER-to-Golgi bypass pathway. It was reported that glycyrrhizin, a drug for chronic hepatitis patients used in Japan, decreases infectious HCV particle release [90]. Identifying the functions of host factors involved in HCV particle release may provide new opportunities for the development of novel antiviral strategies. 

## Figures and Tables

**Table 1:** Summary of cellular factors involved in HCV particle release discussed in the text.

Protein Names	Role in the Cell	Role in HCV Infection	References
Sar1	COPII-vesicle formation, ER-Golgi trafficking	KD leads to inhibition of release	[22]
Rab1b	COPII-dependent ER to Golgi trafficking	DN expression reduces release	[42]
COPII	Traffic from ER to Golgi	Budding from ER	[23]
Clathrin	Vesicle formation at TGN, endosomes, and plasma membrane	KD decreases extracellular infectivity and RNA amount	[61]
AP-1A	TGN to recycling endosome bidirectional transport	KD decreases extracellular infectivity and RNA amount	[22,61,62]
GGA2	TGN to recycling endosome transport	KD decreases extracellular infectivity	[21]
Rab 11A	TGN to recycling endosome transport	KD results in accumulation of core at the Golgi	[22]
Rab 13	TGN to recycling endosome transport	DN leads to inhibition of release	[21]
VPS4A	Late steps of MVB biogenesis, membrane fission	KD or DN expression reduces extracellular infectivity	[24,26]
VPS4B	Late steps of MVB biogenesis, membrane fission	KD or DN expression reduces extracellular infectivity	[25,26]
CHMP4B	Subunit of ESCRT-III complex, membrane fission	KD or DN expression reduces extracellular infectivity	[25,26]
TSG101	Subunit of ESCRT-0, cargo sorting	KD reduces extracellular infectivity	[25]
Alix	MVB biogenesis	KD reduces extracellular infectivity	[25,66]
Itch	HECT-type E3 ubiquitin ligase	KD reduces extracellular infectivity	[24]

KD, knockdown; DN, dominant negative.
